# IMPACT OF AN INTENSIVE MULTIDOMAIN LIFESTYLE INTERVENTION ON DEFICIT ACCUMULATION FRAILTY INDICES

**DOI:** 10.1093/geroni/igz038.2874

**Published:** 2019-11-08

**Authors:** Felicia Simpson, Nicholas M Pajewski, Alain M Bertoni, Frank Ingram, Barbara M Nicklas, Stephen Kritchevsky, Daniel Ojeranti, Mark M Espeland

**Affiliations:** 1 Winston-Salem State University, Winston-Salem, North Carolina, United States; 2 Wake Forest School of Medicine, Winston-Salem, North Carolina, United States

## Abstract

Background: Type 2 diabetes and obesity increase accumulation of health deficits over time and may accelerate biological aging. It is unknown whether multidomain lifestyle interventions can mitigate against this. Methods: Within a large, randomized controlled clinical trial of intensive lifestyle intervention (ILI) including caloric restriction, increased physical activity, dietary counseling, and risk factor monitoring compared with diabetes support and education (DSE) we examined the trajectory of frailty across 8 years. We used two complementary frailty index (FI) definitions, one modeled on work in the Systolic Blood Pressure Intervention Trial; the other including additional deficits related to aging with obesity and type 2 diabetes mellitus. Differences between intervention groups and the consistency of these across clinical subgroups were assessed with re-randomization tests. Results: Data from 4859 adults (45-76 years at baseline, 59% female) were analyzed. Random assignment to ILI was associated with lower FI scores throughout 8 years of follow-up (p<0.001), over which time mean differences between intervention groups averaged 5.8% and 5.4% for the two indices. At year 8, the percentages of participants categorized as frail (FI>0.21) were lower among ILI (39.8% and 54.5%) compared with DSE (42.7% and 60.9%) for the two indices (both p<0.001). Intervention benefits were relatively greater for individuals who were older, not obese, and without history of cardiovascular disease at baseline. Conclusions: Eight years of multidomain lifestyle intervention slows the accumulation of health deficits over time in overweight or obese adults with type 2 diabetes.

